
JOURNAL INFORMATION
==============================
NLM Title Abbreviation: ScientificWorldJournal
Journal ID: ScientificWorldJournal
NLM Journal ID: 101131163
Journal ID: TSWJ

The Scientific World Journal

ISSN: 2356-6140
EISSN: 1537-744X
Publisher: Wiley

ARTICLE INFORMATION
==============================
PMCID: PMC6083968
PMID: 30147574
DOI: 10.1100/tsw.2001.212
Article ID: 363596
Article version: 1
Subjects: Short Report

Regulation of X Chromosome-Linked Inhibitor of Apoptosis Protein (XIAP) in Kainic Acid Induced Neuronal Cell Death in the Rat Hippocampus

Korhonen Laura 1 *
Belluardo Natale 2
Lindholm Dan 1
1 Department of Neuroscience, Neurobiology, Uppsala university, Biomedical Centre, Box 587, S-751 23 Uppsala, Sweden
2 Institute of Human Physiology, Faculty of Medicine, University of Palermo, I-951 25 Palermo, Italy
*Laura Korhonen: laura.korhonen@neuro.uu.se
Publication date: 2001
Electronic publication date: 2001 Dec 12
Volume: 1
First page: 97
Last page: 97
Copyright: Copyright © 2001 Laura Korhonen et al.
Copyright year: 2001
License: This is an open access article distributed under the Creative Commons Attribution License, which permits unrestricted use, distribution, and reproduction in any medium, provided the original work is properly cited.
License URL: https://creativecommons.org/licenses/by/3.0/

Funding: Swedish Cancer Foundation European Commission Stiftelsen för Strategisk Forskning Swedish Society for Medical Research

==============================
Miami *Nature Biotechnology* Short Reports
*TheScientificWorld* (2001) 1 (S3), 97SR
ISSN 1532-2246; DOI 10.1100/tsw.2001.212

### REGULATION OF X CHROMOSOME-LINKED INHIBITOR OF APOPTOSIS PROTEIN (XIAP) IN KAINIC ACID INDUCED NEURONAL CELL DEATH IN THE RAT HIPPOCAMPUS

Laura Korhonen[1*], Natale Belluardo[2], and Dan Lindholm[1]

1. Department of Neuroscience, Neurobiology, Uppsala university, Biomedical Centre, Box 587, S-751 23 Uppsala, Sweden; 2. Institute of Human Physiology, Faculty of Medicine, University of Palermo, I-951 25 Palermo, Italy
* Laura.Korhonen@neuro.uu.se

**INTRODUCTION.** Inhibitor of apoptosis protein (IAP) family consists of several anti-apoptotic proteins conserved among species. The IAPs have a well-conserved motif of approximately 65 residues, called the baculovirus inhibitory repeat (BIR) (1). Baculovirus and drosophila IAPs have two, but most IAPs contain three BIR domains. Most of the IAPs also have a C-terminal RING domain which consists of conserved amino acids with zinc binding capacity. XIAP is one of the five known human IAPs and it binds directly and inhibits the activity of caspases (2). The BIR2 domain in XIAP is sufficient to mediate this inhibition (3). However little is known about the presence and function of XIAP in the nervous system.

**METHOD.** In this study, we have investigated the expression and distribution of XIAP mRNA and protein in rat brain using RNase protection assay in addition to Western blotting and immunohistochemistry with a specific anti-XIAP antibody. Neuronal cell death upon kainic acid stimulation was investigated using TUNEL method and caspase-3 activation.

**RESULTS.** Two transcripts of XIAP mRNA are present in rat brains, suggesting a heterogeneity of XIAP in the carboxyterminal region. Using a specific anti-XIAP antibody, XIAP protein was shown to be expressed in embryonic and adult brains with slightly higher levels in embryonic and neonatal brains compared to adults. There was a widespread distribution of XIAP immunoreactive cells in adult rat brains, among others in hippocampus, cortex and substantia nigra. Using double-labelling, it was shown that both neuronal and glia cells express XIAP. Kainic acid treatment is known to cause widespread neuronal cell death in hippocampus and amygdala. XIAP protein levels were increased in the hippocampus 12th and 24th after kainic acid. The intensity of XIAP staining was increased particularly in the hippocampal CA3 area. In the same area, many TUNEL positive and active caspase-3 positive cells were found. Caspase-3 is activated 24th after kainic acid in vivo and in vitro in cultured embryonic hippocampal neurons upon kainic acid stimulus. In hippocampus, XIAP does not colocalize with markers for cell death raising the possibility that XIAP is expressed in viable cells but not in dying cells. Using cultured embryonic hippocampal neurons, it was shown that XIAP becomes a target for active caspase-3 and that it is degradated into smaller pieces.

**DISCUSSION.** XIAP mRNA is expressed in developing and adult rat brains, and the number of XIAP positive cells and XIAP protein levels were regulated by kainic acid. The largest change in XIAP immunoreactivity was observed in CA3 area, which was particularly vulnerable in this model. Labelling of cells with the TUNEL method and with antibody for active caspase-3 showed that CA3 neurons die in a process resembling apoptosis. Taken together, the results indicate that endogenous XIAP has a protective function in neurons and that the relative levels may determine whether a hippocampal cell will die or not following kainic acid. The present results also demonstrate the cleavage of XIAP in hippocampal neurons in a caspase-3 dependent pathway following an exitotoxic stimulus like kainic acid. This decrease in levels of the anti-apoptotic molecule XIAP by kainic acid may further accelerate cell death in vulnerable neurons and regions of hippocampus. A similar mechanism has recently been proposed for the Fas induced death of immune cells after XIAP cleavage (4). It remains to be studied whether XIAP levels are also regulated in other types of brain injuries and in neurodegeneration.

**ACKNOWLEDGEMENT.** This study was supported by the Swedish Cancer Foundation, a European Commission Biotech grant, the Swedish Foundation of Strategic Research, the Swedish Society for Medical Research, and the University of Palermo.

**REFERENCES.**

1. Clem, R.J. and Miller, L.K. (1994) Mol. Cell. Biol. 14, 5212-5222
2. Liston, P., Roy, N., Tamai, K., et al. (1996) Nature 379, 349-353
3. Deveraux, Q.L., Roy, N., Stennicke, H.R., et al. (1998) EMBO J. 15, 2215-2223
4. Deveraux, Q.L., Leo, E., Stennicke, H.R., et al. (1999) EMBO J. 18, 5242-5251
